# Consolidative versus salvage stereotactic ablative radiotherapy to the primary lung tumor in stage IV non–small cell lung cancer

**DOI:** 10.3389/fonc.2026.1758011

**Published:** 2026-07-15

**Authors:** Lisi Sun, Lulu Wang, Lina Yang, Wei Zhou, Yongzhong Wu, Dan Tao

**Affiliations:** 1 Department of Radiation Oncology, Chongqing University Cancer Hospital, Chongqing, China; 2 Chongqing Key Laboratory of Translational Research for Cancer Metastasis and Individualized Treatment, Chongqing University Cancer Hospital, Chongqing, China

**Keywords:** consolidation therapy, local progression-free survival, non–small cell lung cancer, radiation pneumonitis, stereotactic ablative radiotherapy

## Abstract

**Background:**

The optimal timing of stereotactic ablative radiotherapy (SABR) to the primary lung tumor—whether delivered as consolidative therapy or as salvage therapy —remains undefined in stage IV non–small cell lung cancer (NSCLC). This study compared the efficacy and safety of these two strategies.

**Methods:**

We retrospectively analyzed 90 patients with stage IV NSCLC who received primary lung tumor SABR between October 2020 and November 2025. Patients were categorized into consolidative SABR (n=64) and salvage SABR (n=26). Endpoints included local progression-free survival (LPFS), distant metastasis-free survival (DMFS), and overall survival (OS). Competing risk analyses and multivariable Cox regression were applied. Sensitivity analyses excluding *EGFR/ALK*-positive patients were performed.

**Results:**

Median follow-up was 38.8 months. Consolidative and salvage SABR showed comparable LPFS (1-year rate: 93.8% vs. 84.6%; P = 0.848), DMFS (75.0% vs. 61.5%; P = 0.806), and OS (96.2% vs. 77.6%; P = 0.775). In multivariable analysis, *EGFR/ALK* mutation was independently associated with improved LPFS (HR 0.32, 95% CI 0.16–0.65; P = 0.002), DMFS (HR 0.46, 95% CI 0.25–0.83; P = 0.010), and OS (HR 0.43, 95% CI 0.20–0.93; P = 0.032); oligometastasis predicted superior OS (HR 0.30, 95% CI 0.12–0.75; P = 0.009). SABR timing was not associated with any endpoint. Sensitivity analyses restricted to *EGFR/ALK*-negative patients (n=29) consistently showed no association between SABR timing and survival. Grade ≥2 radiation pneumonitis occurred in 12.2% of patients, with no between-group difference (P = 1.000).

**Conclusions:**

In stage IV NSCLC, consolidative and salvage SABR to the primary tumor achieved comparable local control, distant progression, and overall survival, with analogous safety profiles. These findings indicate that the timing of primary tumor ablation does not independently dictate long-term oncologic outcomes in the context of effective modern systemic therapy. The decision between early consolidation and deferred salvage may be guided by tumor anatomy, anticipated toxicity, and patient preference rather than by anticipated survival advantages. Future prospective trials should adopt non-inferiority frameworks and prioritize composite endpoints—including toxicity, quality of life, and cost-effectiveness—to definitively optimize patient selection and sequencing for this strategy.

## Background

Lung cancer is the leading cause of cancer-related mortality worldwide, with non-small cell lung cancer (NSCLC) constituting approximately 85% of all cases (1). More than one-third of patients present with stage IV disease at diagnosis (2). Systemic therapies, particularly targeted agents and immunotherapy, have markedly improved survival outcomes, recently (3–6). In Asian populations, where sensitizing *EGFR* mutations and *ALK* rearrangements occur in over 50% of lung adenocarcinomas, targeted therapy constitutes the backbone of first-line treatment (7). Nonetheless, acquired resistance is still inevitable, and progression at the primary lung lesion persists as a dominant pattern of failure (8–11).

The role of stereotactic ablative radiotherapy (SABR) in the salvage setting for oligo-progressive NSCLC is well established, enabling continuation of effective systemic therapies (12, 13) and endorsed by current guidelines (14). By contrast, evidence for consolidative local radiotherapy remains heterogeneous and phase III data are limited (14–19). Landmark trials such as SINDAS have demonstrated that upfront SABR to all lesions improved both progression-free survival (PFS) and overall survival (OS) in *EGFR*-mutated oligometastatic NSCLC (20, 21). Notably, these studies address distinct clinical scenarios—salvage after systemic therapy failure or comprehensive ablation of all visible lesions—leaving a critical gap regarding the optimal timing of primary tumor ablation in the broader advanced NSCLC population.

To address this gap, we conducted a retrospective study directly comparing consolidative versus salvage SABR directed exclusively at the primary lung tumor. We hypothesized that these two strategies might yield differential patterns of failure and survival outcomes and sought to provide comparative evidence to inform individualized treatment decisions.

## Methods

### Patient selection

We retrospectively reviewed patients with NSCLC who received lung SABR at the Department of Radiation Oncology, Chongqing University Cancer Hospital, between October 2020 to December 2025. Inclusion criteria were: (1) histologically confirmed stage IV NSCLC per the 8th edition of the American Joint Committee on Cancer (AJCC) Staging System; (2) receipt of SABR or hypofractionated radiotherapy to the primary lung tumor during first-line systemic therapy or its maintenance phase; (3) Eastern Cooperative Oncology Group Performance Status (ECOG-PS) 0–2. Exclusion criteria were: (1) history of two or more primary malignancies; (2) receipt of more than one line of systemic therapy prior to SABR; (3) re-irradiation or conventionally fractionated thoracic radiotherapy; (4) stage III disease; (5) loss to follow-up. This study was approved by the Institutional Ethics Committee of Chongqing University Cancer Hospital (CZLL2025-059-001), and the requirement for informed consent was waived due to its retrospective nature.

### Treatment

All patients received first-line systemic therapy in accordance with prevailing clinical guidelines. Consolidative SABR was administered to the primary lung tumor before disease progression, whereas salvage SABR was delivered to the primary lung tumor that presented as a site of oligo-progression. Local therapy to metastatic lesions was permitted at the discretion of the multidisciplinary team. Oligo-metastasis was defined as the presence of ≤5 metastatic lesions in ≤3 organs. Oligo-progression was defined as progression of ≤5 individual lesions (new or enlarging) during first-line systemic therapy (13).

### SABR delivery

Patients underwent four-dimensional computed tomography (4D-CT) simulation with intravenous contrast. The gross tumor volume (GTV) was delineated to encompass the primary lung tumor, an internal target volume (ITV) was generated from the 4D-CT dataset, and the planning target volume was created by expanding the ITV with a 5-mm isotropic margin. SABR regimens were prescribed by senior radiation oncologists based on tumor size, location (central vs. peripheral), and proximity to organs at risk. Treatment was delivered using the EDGE™ linear accelerator (Varian Medical Systems, Palo Alto, CA) once daily, Monday through Friday. Hypofractionated regimens (e.g., 4 Gy per fraction) were employed for ultra-central tumors.

### Follow-up

Laboratory tests, contrast-enhanced CT or positron emission tomography-CT (PET-CT) if necessary, were recommended every 6–8 weeks, and subsequently every 3–6 months or as clinically indicated.

### Endpoints

The primary endpoints were local progression-free survival (LPFS), secondary endpoints included distant metastasis-free survival (DMFS), overall survival (OS) and incidence of Grade ≥2 radiation pneumonitis. LPFS was defined as the interval from SABR to local progression at the treated primary site or death from any cause, whichever occurred first. DMFS was defined as the interval from SABR to distant metastasis or death from any cause. OS was calculated from the initiation of first-line systemic therapy to death from any cause. Patients without events were censored at the date of last follow-up (Lock Date: May 1, 2026). Treatment response was assessed according to the Response Evaluation Criteria in Solid Tumors (RECIST) version 1.1. The best overall response was categorized as a complete response (CR), partial response (PR), stable disease (SD), or progressive disease (PD). Adverse events were graded per Common Terminology Criteria for Adverse Events (CTCAE) version 5.0. Radiation pneumonitis (RP) assessment window was defined as the 90-day period following completion of SABR.

### Statistical analysis

Continuous and categorical variables were compared using the Mann–Whitney U test and Chi-square or Fisher’s exact test, respectively. Survival probabilities were estimated using the Kaplan–Meier method, with between-group comparisons by the log-rank test; median follow-up was estimated by the reverse Kaplan–Meier method. For competing risk analyses, cumulative incidence of local progression and distant metastasis was modeled using the Fine–Gray subdistribution hazard model with death as a competing risk, and compared between groups using Gray tests.

Prognostic factor analysis was performed using Cox proportional hazards regression. The proportional hazards assumption was assessed using Schoenfeld residual tests. For LPFS, the global test was marginally significant (P = 0.048), driven by a time-varying effect of oligometastatic status (P = 0.002). Consequently, the multivariable LPFS model was stratified by metastatic burden. Standard Cox models were used for DMFS and OS. The primary multivariable model included SABR timing, *EGFR/ALK* mutation status, metastatic burden, best response to first-line treatment, and BED10 (analyzed as a continuous variable per 10-Gy increment), selected *a priori* based on clinical relevance. Age showed no association with LPFS in univariate analysis and was well balanced between groups; sex, smoking status, and GTV showed univariate significance but were excluded from the primary model to avoid overfitting given limited events per variable.

Propensity score matching was attempted using a 1:1 nearest-neighbor algorithm with a caliper of 0.3 SD of the logit of the propensity score. Post-matching diagnostics revealed worsened balance of *EGFR/ALK* mutation status and first-line treatment response (**Supplementary Table 1**), likely due to limited overlap in the propensity score distribution and the small salvage group (n=26). Consequently, the primary analysis relied on multivariable Cox regression in the full cohort.

Sensitivity analyses excluding *EGFR/ALK*-positive patients to assess generalizability in the chemo-immunotherapy-treated population, and excluded patients treated with hypofractionated radiotherapy (4 Gy per fraction) for ultra-central tumors to evaluate robustness in the standard SABR population. Exploratory subgroup analyses were performed by *EGFR/ALK* status and metastatic burden, with interaction P values calculated using likelihood ratio tests comparing models with and without interaction terms.

For toxicity, exploratory univariate comparisons between patients with and without Grade ≥2 radiation pneumonitis were conducted using Fisher’s exact test and the Mann–Whitney U test, due to the limited number of Grade ≥2 RP events (n=11). Statistical significance was defined as a two-sided P < 0.05. All analyses were conducted using R software (version 4.6.0).

## Results

### Baseline characteristics

**Figure 1:**
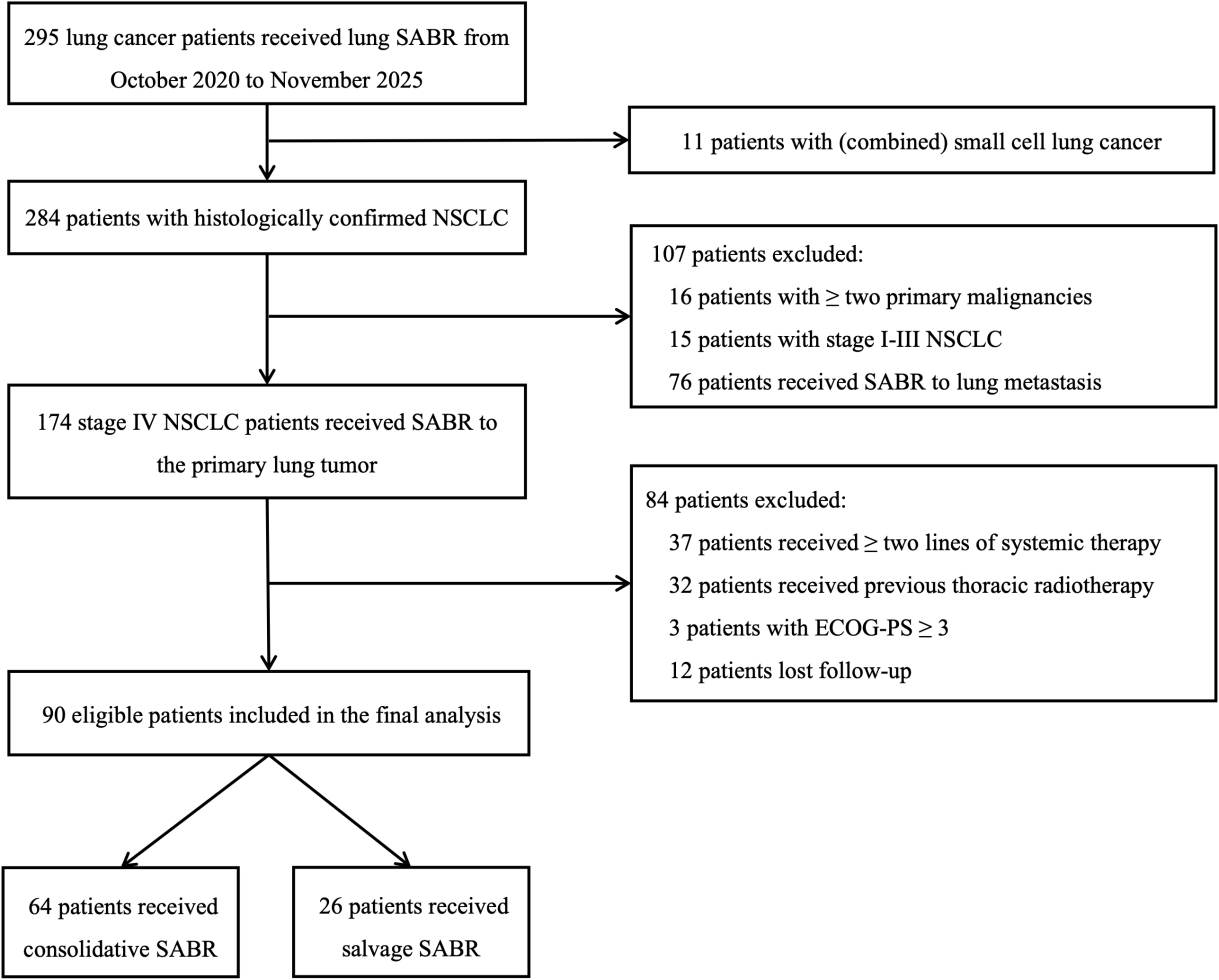
Study flowchart.

A total of 295 patients were assessed for eligibility, and 90 patients were eligible for this study (**Figure 1**). The median age was 62 years (range, 22–88), and 60.0% (n = 54) were male. Adenocarcinoma accounted for 93.3% (n = 84). Forty-two patients (46.7%) had a smoking history. ECOG PS was 0 in 61.1% (n = 55), 1 in 38.9% (n = 32), 2 in 3 patients. Thirty-eight patients (42.2%) had oligometastatic and 52 (57.8%) non-oligometastatic disease. The most frequent metastatic sites were lung (50.0%), bone (48.9%), and brain (37.8%). Sixty patients (66.7%) harbored sensitizing EGFR mutations or ALK fusions. First-line systemic therapies included targeted therapy (66.7%), chemotherapy (42.2%), immunotherapy (31.1%), and bevacizumab (10.0%) (**Table 1**).

**Table 1:** Baseline characteristics of the whole cohort (n=90).

Characteristics	Overall (n=90)	Consolidative SABR (n=64)	Salvage SABR (n=26)	P value
Age (year)				
Median (range)	62.0 (22.0–88.0)	65 (34.0 - 88.0)	61.0 (22.0 - 73.0)	0.950
Sex				
Male	54 (60.0)	34 (53.1)	20 (76.9)	**0.037**
Female	36 (40.0)	30 (46.9)	6 (23.1)	
Smoking history				
Nonsmoker	48(53.3)	36 (56.3)	12 (46.2)	0.384
Smoker	42 (46.7)	28 (43.8)	14 (53.9)	
Histology				
Adenocarcinoma	84 (93.3)	60 (93.8)	24 (92.3)	0.804
Non-adenocarcinoma	6 (6.7)	4 (6.2)	2 (7.7)	
ECOG PS				
0	55 (61.1)	38 (59.4)	17 (65.4)	0.596
≥1	35 (38.9)	26 (40.6)	9 (34.6)	
Driver gene mutation				
*EGFR/ALK* positive ^*^	60 (66.7)	40 (62.5)	20 (76.9)	0.188
Other ^#^	7 (7.8)	6 (9.4)	1 (3.8)	–
Negative	22 (24.4)	17 (26.6)	5 (19.2)	–
Unknown	1 (1.1)	1(1.6)	0 (0)	–
Metastasis burden				
Oligometastatic	38 (42.2)	28 (43.7)	10 (38.5)	0.900
Non-oligometastatic	52 (57.8)	36 (56.3)	16 (61.5)	
Clinical Stage				
IVA	41(45.6)	30 (46.9)	11 (42.3)	0.693
IVB	49(54.4)	34 (53.1)	15 (57.7)	
Metastatic site				
Lung	45 (50.0)	31 (48.4)	14 (53.9)	0.642
Bone	44 (48.9)	34 (53.1)	10 (38.5)	0.207
Brain	34 (37.8)	25 (39.1)	9 (34.7)	0.693
Pleural	17 (18.9)	13 (20.3)	4 (15.4)	0.588
Adrenal	7 (7.8)	6 (9.4)	1 (3.9)	0.375
Liver	2 (2.2)	1 (1.6)	1 (3.9)	0.505
Other	26 (28.9)	19 (29.7)	7 (26.9)	0.793
First-line systemic therapy				
Targeted therapy	60 (66.7)	41 (64.1)	19 (73.1)	0.411
Chemotherapy	38 (42.2)	29 (45.3)	9 (34.6)	0.352
Immunotherapy	28 (31.1)	21 (32.8)	7 (26.9)	0.584
Bevacizumab	9 (10.0)	9 (14.1)	0 (0.0)	**0.044**
Best response to systemic treatment				
PR	43 (47.8)	33 (51.6)	10 (38.5)	0.259
SD	47 (52.2)	31 (48.4)	16 (61.5)	
Primary tumor location				
Peripheral	50 (55.6)	39 (60.9)	11 (42.3)	0.107
Central	40 (44.4)	25 (39.1)	15 (57.7)	
GTV (cc)				
Median (range)	9.5 (0.8 -92.7)	7.2 (0.8 - 31.4)	12.7 (1.4 - 92.7)	**0.016**
BED10				
Median (range)	100.0(61.6 - 132.0)	100.0 (72.0 - 132.0)	100.0 (61.6 - 132.0)	0.661

Data are presented as median (range) for continuous variables and n (%) for categorical variables. First-line systemic therapy categories are not mutually exclusive; patients could receive multiple modalities concurrently. Bold values indicate statistically significant differences (P < 0.05).

*: *EGFR/ALK* positive included *EGFR 19* deletion, *EGFR 21 L858R*, *EGFR-T790M*, *EGFR-L861Q*, *EGFR-G719X*, *EML4-ALK* rearrangemen.

#: Other mutations included Kirsten rat sarcoma viral oncogene (*KRAS*), BRaf proto-oncogene, serine/threonine kinase (*BRAF*), transfection proto-oncogene gene (*RET*), and human epidermal growth factor receptor 2 (*HER2*).

SABR, stereotactic ablative radiotherapy; EGFR, epidermal growth factor receptor; ALK, anaplastic lymphoma kinase; PR, partial response; SD, stable disease; ECOG-PS, Eastern Cooperative Oncology Group Performance Status; GTV, gross tumor volume; BED10, biologically effective dose with α/β=10.

### Primary lung tumor SABR details

Consolidative SABR was administered in 64 patients (71.1%) and salvage SABR in 27 (28.9%). The median interval from initiation of first-line systemic therapy to SABR was 6.8 months, 5.6 months in the consolidative group and 10.3 months in the salvage group (p = 0.001). The most frequently employed SABR schedules were 10 Gy in 5 fractions with a median biological equivalent dose 10 (BED10) of 100.0 Gy (range, 61.6–132.0). Seven patients with ultra-central tumors received hypofractionated regimens (e.g., 4 Gy per fractions). Clinical characteristics were well balanced between the two groups except for a higher proportion of males in the salvage group (76.9% vs. 53.1%, P = 0.037), a larger median GTV in the salvage group (12.7 cc vs. 7.2 cc, P = 0.016), and exclusive use of bevacizumab in the consolidative group (14.1% vs. 0%, P = 0.044) (**Table 1**).

### Survival outcomes

As of the data cutoff (May 1, 2026), the median follow-up duration was 38.8 months (95% CI, 33.9–46.4). For the entire cohort, median LPFS, DMFS, and OS were 43.3 months (95% CI, 32.4–54.2), 29.4 months (95% CI, 17.3–41.5), and 53.1 months (95% CI, 41.9–not reached), respectively. The 1-year and 2-year rates were 90.0% and 80.7% for LPFS, 70.0% and 56.1% for DMFS, and 96.7% and 87.4% for OS (**Table 2**).

**Table 2:** Survival outcomes in the whole cohort and by SABR timing.

Endpoint	Time point	Whole cohort (n=90)	Consolidative SABR (n=64)	Salvage SABR (n=26)	P value
LPFS	1-year rate, % (95% CI)	90.0 (83.7–96.3)	93.8 (88.0–99.9)	84.6 (71.8–99.7)	0.848
	2-year rate, % (95% CI)	80.7 (72.5–88.9)	85.9 (77.8–94.9)	73.1 (57.9–92.3)	
	Median (95% CI), months	43.3 (32.4–54.2)	42.7 (36.7–48.7)	52.6 (22.0–83.2)	
DMFS	1-year rate, % (95% CI)	70.0 (59.5–78.3)	75.0 (65.1–83.4)	61.5 (45.4–83.4)	0.806
	2-year rate, % (95% CI)	56.1 (45.1–65.8)	58.6 (47.5–72.2)	50.0 (34.0–73.4)	
	Median (95% CI), months	29.4 (17.3–41.5)	29.4 (23.0–36.8)	19.1 (9.0–48.7)	
OS	1-year rate, % (95% CI)	96.7 (90.0–98.9)	96.2 (89.0–100.0)	77.6 (61.2–98.3)	0.775
	2-year rate, % (95% CI)	87.4 (78.3–92.9)	90.6 (83.7–98.1)	84.6 (71.8–99.7)	
	3-year rate, % (95% CI)	70.6 (58.0–80.0)	67.7 (55.5–82.6)	77.6 (61.2–98.3)	
	Median (95% CI), months	53.1 (41.9–NR)	59.6 (33.4–85.8)	53.1 (52.1–54.1)	

P values were calculated using the log-rank test comparing survival curves between the consolidative and salvage SABR groups. The reverse Kaplan–Meier method was used to estimate median follow-up. NR indicates not reached.

SABR, stereotactic ablative radiotherapy; LPFS, local progression-free survival; DMFS, distant metastasis-free survival; OS, overall survival; CI, confidence interval.

### Survival outcomes by SABR timing

**Figure 2:**
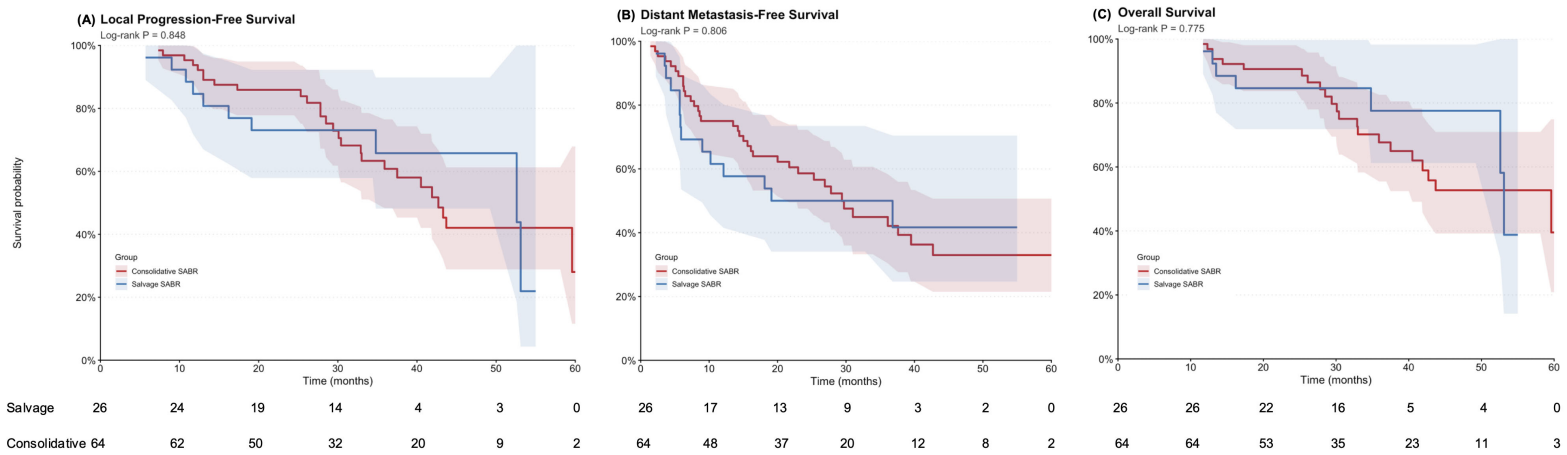
Kaplan-Meier survival curves. Kaplan–Meier survival curves for **(A)** local progression-free survival (LPFS), **(B)** distant metastasis-free survival (DMFS), and **(C)** overall survival (OS). Survival probabilities are shown for the consolidative SABR group (red) and salvage SABR group (blue). P values were calculated using the log-rank test. Numbers at risk are shown below each plot. SABR, stereotactic ablative radiotherapy.

Patients receiving consolidative SABR and salvage SABR showed comparable survival outcomes. Median LPFS was 42.7 months (95% CI, 36.7–48.7) in the consolidative group versus 52.6 months (95% CI, 22.0–83.2) in the salvage group (P = 0.848). Median DMFS was 29.4 months (95% CI, 23.0–36.8) versus 19.1 months (95% CI, 9.0–48.7) (P = 0.806). Median OS was 59.6 months (95% CI, 33.4–85.8) versus 53.1 months (95% CI, 52.1–54.1) (P = 0.775). The 1-year, 2-year, and 3-year OS rates were 96.2%, 90.6%, and 67.7% in the consolidative group versus 77.6%, 84.6%, and 77.6% in the salvage group (**Figure 2**; **Table 2**).

### Competing risk analysis

**Figure 3:**
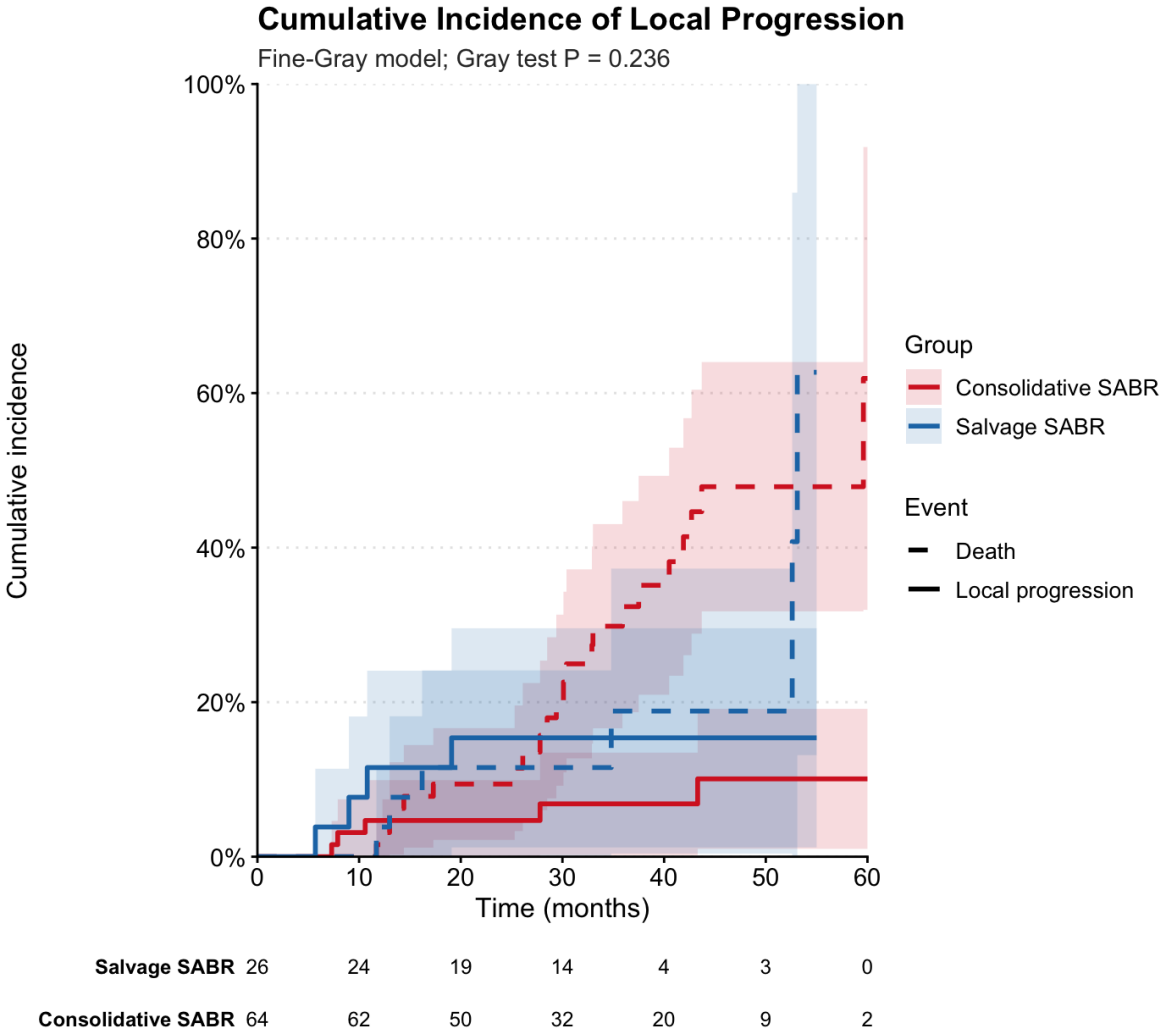
Cumulative incidence of local progression (Fine–Gray model). Cumulative incidence function (CIF) curves for local progression (solid lines) and death (dashed lines) in the consolidative SABR group (red) and salvage SABR group (blue). Death was treated as a competing risk. The Gray test P value is shown. SABR, stereotactic body radiotherapy.

In the Fine–Gray competing risk model, the cumulative incidence of local progression did not differ between consolidative and salvage SABR at 20 months (4.7% vs. 15.4%) or 40 months (6.8% vs. 15.4%; Gray test χ² = 1.40, P = 0.236) (**Figure 3**; **Supplementary Table 2**). Similarly, the cumulative incidence of distant metastasis was comparable between groups (Gray test χ² = 0.43, P = 0.513; **Supplementary Table 3**). In the Fine–Gray multivariable model for local progression, SABR timing remained unassociated with local failure (subdistribution HR 0.43, 95% CI 0.13–1.44; P = 0.107; **Supplementary Table 4**).

### Prognostic factor analysis

**Figure 4:**
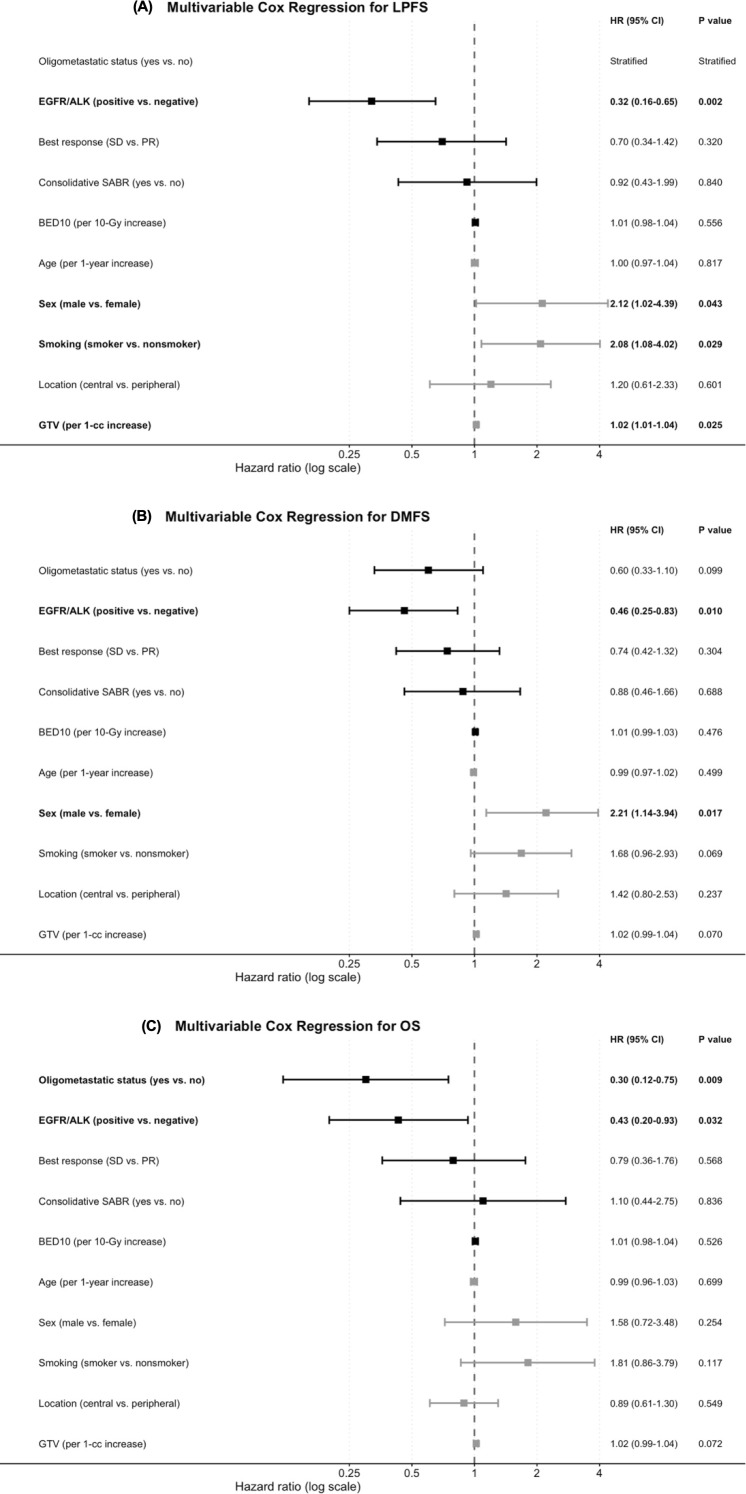
Forest plot of multivariable Cox regression for LPFS, DMFS, and OS. **(A)** LPFS, **(B)** DMFS, **(C)** OS. Hazard ratios (squares) and 95% confidence intervals (horizontal lines) are shown for the prespecified multivariable models. The vertical dashed line indicates the null value (HR = 1). Variables with univariate significance but excluded from the primary model due to collinearity or limited events per variable (sex, smoking history, GTV) are shown in gray for reference. LPFS, local progression-free survival; DMFS, distant metastasis-free survival; OS, overall survival; HR, hazard ratio; CI, confidence interval; *EGFR*, epidermal growth factor receptor; *ALK*, anaplastic lymphoma kinase; SD, stable disease; PR, partial response; BED10, biologically effective dose with α/β=10; GTV, gross tumor volume.

For LPFS, *EGFR/ALK* positivity was the only independent protective factor (adjusted HR 0.32, 95% CI 0.16–0.65; P = 0.002), whereas consolidative SABR timing (adjusted HR 0.92, 95% CI 0.43–1.99; P = 0.840) was not significantly associated with improved local control (**Table 3**; **Figure 4A**). For DMFS, *EGFR/ALK* positivity was the sole independent protective factor (adjusted HR 0.46, 95% CI 0.25–0.83; P = 0.010). SABR timing was not significant (adjusted HR 0.88, 95% CI 0.46–1.66; P = 0.688) (**Table 4**; **Figure 4B**). For OS, oligometastatic status (adjusted HR 0.30, 95% CI 0.12–0.75; P = 0.009) and EGFR/ALK positivity (adjusted HR 0.43, 95% CI 0.20–0.93; P = 0.032) were independent predictors. SABR timing was not associated with OS (adjusted HR 1.10, 95% CI 0.44–2.75; P = 0.836) (**Table 5**; **Figure 4C**).

**Table 3:** Univariate and multivariate analysis of clinical factors associated with LPFS.

Variable	Univariable analysis			Multivariable analysis		
	HR	95% CI	p	HR	95% CI	p
Oligometastatic status (yes vs. no)	0.64	0.32 - 1.26	0.194	Stratified§	–	–
EGFR/ALK (positive vs. negative)	0.35	0.18 - 0.67	**0.002**	0.32	0.16 - 0.65	**0.002**
Best response (SD vs. PR)	0.82	0.43 - 1.56	0.538	0.70	0.34 - 1.42	0.320
Consolidative SABR (yes vs. no)	0.93	0.45 - 1.94	0.848	0.92	0.43 - 1.99	0.840
BED10	1.01	0.98 - 1.04	0.463	1.01	0.98 - 1.04	0.556
Age	1.00	0.97 - 1.04	0.817	–	–	–
Sex (male vs. female)	2.12	1.02 - 4.39	**0.043**	–	–	–
Smoking (smoker vs. nonsmoker)	2.08	1.08 - 4.02	**0.029**	–	–	–
Location (central vs. peripheral)	1.20	0.61 - 2.33	0.601	–	–	–
GTV	1.02	1.01 - 1.04	**0.025**	–	–	–

**§** The multivariable model was stratified by oligometastatic status due to violation of the proportional hazards assumption (Schoenfeld residual test P = 0.002); therefore, no hazard ratio is presented for this variable. Sex, smoking status were excluded from the primary multivariable model due to strong collinearity with *EGFR/ALK* status and to avoid overfitting given limited events per variable. GTV was excluded from the primary multivariable model to preserve statistical efficiency given limited LPFS events (n=37). Sensitivity analyses including GTV produced qualitatively identical inferences for SABR timing and *EGFR/ALK* status (data not shown).

LPFS, local progression-free survival; HR, hazard ratio; CI, confidence interval; SABR, stereotactic ablative radiotherapy; *EGFR*, epidermal growth factor receptor; *ALK*, anaplastic lymphoma kinase; BED10, biologically effective dose with α/β=10; SD, stable disease; PR, partial response; GTV, gross tumor volume.

Bold values denote statistically significant differences between the consolidative SABR and salvage SABR groups (P < 0.05).

**Table 4:** Univariate and multivariate analysis of clinical factors associated with DMFS.

Variable	Univariable analysis			Multivariable analysis		
	HR	95% CI	p	HR	95% CI	p
Oligometastatic status (yes vs. no)	0.72	0.40 - 1.27	0.255	0.60	0.33 - 1.10	0.099
EGFR/ALK (positive vs. negative)	0.517	0.29 - 0.91	**0.022**	0.46	0.25 - 0.83	**0.010**
Best response (SD vs. PR)	0.80	0.45 - 1.36	0.381	0.74	0.42 - 1.32	0.304
Consolidative SABR (yes vs. no)	0.93	0.50 - 1.72	0.807	0.88	0.46 - 1.66	0.688
BED10	1.01	0.98 - 1.03	0.714	1.01	0.99 - 1.03	0.476
Age	0.99	0.97 - 1.02	0.499	–	–	–
Sex (male vs. female)	2.21	1.14 - 3.94	**0.017**	–	–	–
Smoking (smoker vs. nonsmoker)	1.68	0.96 - 2.93	0.069	–	–	–
Location (central vs. peripheral)	1.42	0.80 - 2.53	0.237	–	–	–
GTV	1.02	0.99 - 1.04	0.070	–	–	–

The multivariable model included SBRT timing, EGFR/ALK mutation status, metastatic burden, first-line treatment response, and BED10, selected *a priori* based on clinical relevance. Sex, smoking history, and GTV showed univariate significance or trends but were excluded from the primary model to avoid overfitting given limited events per variable.

DMFS, distant metastasis-free survival; HR, hazard ratio; CI, confidence interval; SABR, stereotactic ablative radiotherapy; *EGFR*, epidermal growth factor receptor; *ALK*, anaplastic lymphoma kinase; BED10, biologically effective dose with α/β=10; SD, stable disease; PR, partial response; GTV, gross tumor volume.

Bold values denote statistically significant differences between the consolidative SABR and salvage SABR groups (P < 0.05).

**Table 5:** Univariate and multivariate analysis of clinical factors associated with OS.

Variable	Univariable analysis			Multivariable analysis		
	HR	95% CI	p	HR	95% CI	p
Oligometastatic status (yes vs. no)	0.40	0.17 - 0.91	**0.028**	0.30	0.12 - 0.75	**0.009**
EGFR/ALK (positive vs. negative)	0.51	0.24 - 1.06	0.071	0.43	0.20 - 0.93	**0.032**
Best response (SD vs. PR)	1.06	0.51 - 2.20	0.867	0.79	0.36 - 1.76	0.568
Consolidative SABR (yes vs. no)	1.13	0.48 - 2.67	0.775	1.10	0.44 - 2.75	0.836
BED10	1.01	0.98 - 1.04	0.620	1.01	0.98 - 1.04	0.526
Age	0.99	0.96 - 1.03	0.699	–	–	–
Sex (male vs. female)	1.58	0.72 - 3.48	0.254	–	–	–
Smoking (smoker vs. nonsmoker)	1.81	0.86 - 3.79	0.117	–	–	–
Location (central vs. peripheral)	0.89	0.61 - 1.30	0.549	–	–	–
GTV	1.02	0.99 - 1.04	0.072	–	–	–

The multivariable model included SBRT timing, *EGFR/ALK* mutation status, metastatic burden, first-line treatment response, and BED10, selected *a priori* based on clinical relevance. Sex, smoking history, and GTV showed univariate significance or trends but were excluded from the primary model to avoid overfitting given limited events per variable.

OS, overall survival; HR, hazard ratio; CI, confidence interval; SABR, stereotactic ablative radiotherapy; *EGFR*, epidermal growth factor receptor; *ALK*, anaplastic lymphoma kinase; BED10, biologically effective dose with α/β=10; SD, stable disease; PR, partial response; GTV, gross tumor volume.

Bold values denote statistically significant differences between the consolidative SABR and salvage SABR groups (P < 0.05).

### Sensitivity analyses

In the sensitivity analysis restricted to *EGFR/ALK*-negative patients (n=29), consolidative SABR remained unassociated with LPFS (stratified adjusted HR 1.11, 95% CI 0.24–5.14; P = 0.873), DMFS (adjusted HR 0.64, 95% CI 0.21–1.93; P = 0.435), and OS (adjusted HR 0.69, 95% CI 0.18–2.66; P = 0.598), consistent with the primary model.

Sensitivity analysis excluding patients treated with 4 Gy per fraction (n=7) was performed among those who received standard SABR (n=83). In this restricted cohort, consolidative and salvage SABR remained comparable across all endpoints: LPFS (P = 0.658), DMFS (P = 0.636), and OS (P = 0.976). The cumulative incidence of local progression did not differ between groups (Gray test P = 0.260). In multivariable analysis, SABR timing remained unassociated with LPFS (stratified adjusted HR 0.82, 95% CI 0.37–1.83; P = 0.631), DMFS (adjusted HR 0.82, 95% CI 0.43–1.56; P = 0.545), and OS (adjusted HR 0.97, 95% CI 0.40–2.35; P = 0.947). *EGFR/ALK* positivity retained its independent protective effect for LPFS (HR 0.31, 95% CI 0.15–0.64; P = 0.001), DMFS (HR 0.42, 95% CI 0.22–0.80; P = 0.006), and OS (HR 0.39, 95% CI 0.18–0.85; P = 0.023). Oligometastatic status predicted better OS (HR 0.30, 95% CI 0.13–0.69; P = 0.009) and showed a borderline association with DMFS (HR 0.54, 95% CI 0.29–1.00; P = 0.050). These findings were materially unchanged from the primary model.

### Subgroup analyses

Exploratory subgroup analyses for LPFS by SABR timing showed comparable effect estimates across *EGFR/ALK*-positive (HR 0.72, 95% CI 0.30–1.70; P = 0.456) and *EGFR/ALK*-negative (HR 1.25, 95% CI 0.40–3.90; P = 0.698) strata (interaction P = 0.385). Similarly, no differential effect was observed between oligometastatic (HR 0.65, 95% CI 0.25–1.70; P = 0.378) and non-oligometastatic (HR 1.35, 95% CI 0.50–3.65; P = 0.545) subgroups (interaction P = 0.256) (**Supplementary Figure 1**).

### Radiation induced toxicities

Grade ≥2 radiation pneumonitis occurred in 11 patients (12.2%), with comparable incidence between the consolidative and salvage SABR groups (10.9% [7/64] vs. 15.4% [4/26]; P = 1.000 by Fisher’s exact test). The median time to onset was 3.5 months (range, 2.6–4.8). All cases were managed with corticosteroids. There were no Grade 4–5 events, and no patient died from pulmonary toxicity. Grade ≥2 radiation esophagitis occurred in three patients (3.3%, two received consolidative and one received salvage SABR), which resolved with supportive care. No other Grade ≥3 toxicities were observed.

Exploratory univariate analyses showed no significant differences in BED10 (P = 0.073), fraction size (P = 0.747), or GTV (P = 0.219) between patients with and without Grade ≥2 RP. Central tumor location showed a numerical increase in risk (17.5% vs. 8.0%) but did not reach statistical significance (P = 0.124) (**Supplementary Table 5**).

## Discussion

The present study reveals that consolidative and salvage SABR directed at the primary lung tumor achieved statistically comparable local control, distant metastasis control, and overall survival in stage IV NSCLC after a median follow-up of 38.8 months. This null association persisted across multivariable Cox regression, Fine–Gray competing risk models, subgroup analyses by metastatic burden and sensitivity analyses restricted to *EGFR/ALK*-negative patients. Multivariable Cox regression yielded hazard ratios approximating the null value across all endpoints (LPFS: HR 0.92, 95% CI 0.43–1.99; DMFS: HR 0.88, 95% CI 0.46–1.66; OS: HR 1.10, 95% CI 0.44–2.75), with wide confidence intervals reflecting the modest sample size, particularly the small salvage group (n = 26). It is important to emphasize, however, that these data demonstrate an absence of statistically significant difference rather than formal statistical equivalence, as the study was not powered for a non-inferiority margin.

The observed median OS of 53.1 months and 2-year OS rate of 87.4% markedly exceed historical benchmarks (22), reflecting a confluence of modern therapeutic advances and stringent selection criteria rather than a spurious cohort effect. Specifically, 66.7% of patients harbored sensitizing *EGFR*/*ALK* alterations and received contemporary TKIs; 96.7% had an ECOG performance status of 0–1. Furthermore, all patients received SABR, yielding a 1-year LPFS of 90.0% that mitigated local progression–related mortality, and the study spanned 2020–2025, when immunotherapy, anti-angiogenic agents, and best supportive care were routinely accessible (23). Because these prognostic factors were balanced between the two SABR timing groups (except for GTV and sex), the overall upward shift in survival does not confound the relative comparison. Rather, the data indicate that *EGFR/ALK* status and metastatic burden—not the radiotherapy calendar—are the dominant determinants of prognosis in this setting.

Notably, the salvage group exhibited a larger median GTV (12.7 cc vs. 7.2 cc; P = 0.016), suggesting that clinicians instinctively reserved consolidative SABR for smaller lesions while preferring initial systemic therapy alone for bulkier tumors. Despite this unfavorable anatomy in the salvage arm, survival outcomes did not differ. This indicates that effective systemic therapy adequately controls larger lesions, rendering salvage SABR equally effective. This aligns with reports that delaying radiotherapy until after initial systemic therapy response evaluation may optimize the therapeutic ratio (24–26). The observed equivalence is robust even when applied to a salvage population with inherently more challenging disease anatomy.

In competing risk analysis, the cumulative incidence of local progression at 20 months was numerically lower in the consolidative group (4.7% vs. 15.4%), although this did not achieve statistical significance (Gray test P = 0.236). The wide confidence intervals at this time point reflect uncertainty due to the small salvage subgroup, and by 40 months the absolute gap narrowed (6.8% vs. 15.4%). Importantly, this early numerical divergence in local control did not translate into differences in DMFS or OS. This result suggests that any potential early local control advantage of consolidative SABR might be effectively buffered by modern systemic therapy, which suppresses micrometastatic dissemination and mitigates the downstream consequences of local failure.

Our findings contribute a distinct perspective to the current local therapy evidence map. The NRG-LU002 trial demonstrated no PFS or OS benefit from adding local consolidative therapy to maintenance immunotherapy in oligometastatic NSCLC (27), suggesting that indiscriminate early local intervention is not universally beneficial. Conversely, the SINDAS trial (20) and several phase II trials (28–30) reported durable outcomes with comprehensive ablation of all visible lesions. However, these trials could not answer whether the benefit derived from the “early” timing or the “comprehensive” irradiation coverage. The CURB trial validated salvage SABR for oligo-progression but did not compare it against consolidative SABR (13). Our study occupies this precise niche by focusing exclusively on the primary tumor and directly comparing two treatment timings. These data suggest that, as long as SABR is ultimately delivered to the primary site, the specific chronology may not substantially alter overall disease trajectory. When comprehensive irradiation is impractical, deferring primary tumor SABR until oligo-progression appears to be a reasonable strategy. For patients with large GTV, or central tumors threatening critical structures, delaying SABR until systemic cytoreduction may reduce treatment volume and toxicity without compromising survival. Conversely, for symptomatic lesions, early SABR remains appropriate.

Radiotherapy has been shown to potentiate systemic anti-tumor immunity through the abscopal effect, a mechanism that has attracted considerable interest (31). However, whether the abscopal effect is further augmented by early SABR remains unknown. Recent prospective single-arm and phase II studies have reported favorable outcomes with consolidative SABR after first-line chemo-immunotherapy in oligo-residual NSCLC (32, 33). Yet, in our sensitivity analysis restricted to *EGFR/ALK* -negative patients (n = 29), predominantly treated with chemo-immunotherapy, SABR timing was not associated with either OS or DMFS. This suggests that any potential immune-stimulatory benefit of early SABR, when confined to primary tumor ablation alone, does not necessarily translate into measurable survival advantages. Although this analysis is limited by small numbers, it implies that physicians need not feel compelled to routinely integrate early SABR with first-line immunotherapy, thereby avoiding potential uncertainties regarding overlapping toxicities (34). Ongoing clinical trials (NCT03867175, NCT04929041, NCT06313541, jRCTs041200046) should further clarify this issue, as the reproducibility and clinical benefit of the abscopal effect remain to be validated (35, 36).

Exploratory subgroup analyses revealed no differential effect of SABR timing between oligometastatic and non-oligometastatic subgroups (interaction P = 0.256). While these analyses are underpowered and should be considered hypothesis-generating, they converge with previous real-world evidence suggesting that selected non-oligometastatic patients harboring *EGFR* mutations may derive survival benefit from primary tumor radiotherapy (37, 38). Taken together, these data support a shift from rigid numerical cutoffs toward biology-driven decision-making: in patients with controlled extracranial disease, primary tumor SABR—whether early or late—may extend the therapeutic window for subsequent treatments without added toxicity.

Grade ≥2 radiation pneumonitis occurred in 12.2% overall, with comparable rates between groups (10.9% vs. 15.4%; P = 1.000). The absence of inter-group difference in RP incidence, coupled with no incidence of Grade ≥3 toxicities, further supports that SABR timing can be guided by anatomic and dosimetric considerations, patient preference, and systemic therapy continuity, rather than by fear of a closing therapeutic window.

## Limitations

Several limitations warrant acknowledgment. First, the retrospective, single-center design precludes causal inference, and treatment assignment was determined by clinical discretion, introducing selection bias. Second, the stringent inclusion criteria—ECOG PS 0–2, first-line systemic therapy only, and successful SABR delivery—created a highly selected “good-prognosis” cohort that directly contributed to the prolonged survival outcomes; these results should not be extrapolated to unselected or poor-performance-status populations. Third, the salvage group was relatively small (n=26), limiting the statistical power to detect small but clinically relevant survival differences. Nevertheless, the consistency of null results across primary, competing risk, sensitivity, and subgroup analyses strengthens the inference that distinct survival advantages with either timing are unlikely. Fourth, squamous cell carcinoma was underrepresented (6.7%), precluding subgroup analyses. Our findings should not be extrapolated to squamous histologies, where locoregional treatment may play a distinct prognostic role given the historically inferior outcomes with systemic therapy. Finally, limited sample size precluded robust molecular stratification beyond *EGFR/ALK*. Future prospective trials should integrate comprehensive genomic profiling (e.g. KRAS, TP53 alterations) to identify subsets most likely to benefit from consolidative versus salvage SABR.

## Conclusion

In conclusion, this study demonstrates that consolidative and salvage SABR to the primary lung tumor in stage IV NSCLC yield comparable local control, distant progression control, and overall survival, with analogous safety profiles. These findings provide critical real-world evidence that, in the setting of effective modern systemic therapy, the timing of primary tumor ablation can be individualized based on tumor anatomy, toxicity risk, clinical symptoms and patient preference, without compromising long-term survival. Future investigations should adopt non-inferiority frameworks and prioritize composite endpoints—including toxicity, quality of life, and cost-effectiveness—to definitively optimize patient selection and sequencing for this strategy.

## Data Availability

The raw data supporting the conclusions of this article will be made available by the authors, without undue reservation.

## Supplementary material

Supplementary Table 1 Propensity Score Matching (PSM) Balance Diagnostics.

Supplementary Table 2 Fine-Gray Competing Risk Analysis for Cumulative Incidence of Local Progression.

Supplementary Table 3 Fine-Gray Competing Risk Analysis for Cumulative Incidence of distant metastasis.

Supplementary Table 4 Fine-Gray Competing Risk Analysis for Local Progression-Free Survival.

Supplementary Table 5 Exploratory univariate analysis of factors associated with grade ≥2 radiation pneumonitis.

Supplementary Figure 1 Subgroup analyses for LPFS by SABR timing. Hazard ratios (HRs) and 95% confidence intervals (CIs) for the effect of consolidative versus salvage SABR on LPFS within prespecified subgroups. HRs were derived from multivariable Cox regression stratified by subgroup, with the same covariates as the primary model. Interaction P values were calculated using likelihood ratio tests comparing models with and without interaction terms. LPFS, local progression-free survival; SABR, stereotactic ablative radiotherapy; *EGFR*, epidermal growth factor receptor; *ALK*, anaplastic lymphoma kinase.
