# Supplementary material for: Consolidative versus salvage stereotactic ablative radiotherapy to the primary lung tumor in stage IV non–small cell lung cancer
Source: Front Oncol. 2026 Jul 15;16:1758011. doi: 10.3389/fonc.2026.1758011 (PMC13414212; doi:10.3389/fonc.2026.1758011)
Supplement: Supplementary file 1 [file Table1.docx]

**Supplementary Table 1. Propensity Score Matching (PSM) Balance Diagnostics**

| Variable | Before PSM | | | After PSM (1:1) | | |  |
| --- | --- | --- | --- | --- | --- | --- | --- |
|  | Consolidative (n=64) | salvage (n=26) | SMD | Consolidative (n=25) | salvage (n=25) | SMD | SMD Change |
| *EGFR/ALK* positive, % | 62.5 | 68.3 | -0.298 | 44.0 | 76.0 | -0.661 | Worsened |
| Oligometastasis, % | 43.7 | 38.5 | 0.107 | 40 | 40.0 | 0.000 | Improved |
| Best response (SD/PR), % | 51.6 | 38.5 | 0.262 | 8 | 40.0 | -0.640 | Worsened |
| Propensity score | 0.72 | 0.68 | 0.465 | 0.69 | 0.69 | 0.042 | Improved |

PSM was performed using a 1:1 nearest-neighbor algorithm with a caliper of 0.3 SD of the logit of the propensity score. While the propensity score and oligometastatic status achieved adequate balance (SMD <<0.25), *EGFR/ALK* mutation status and first-line treatment response exhibited worsened SMD after matching (|SMD| > 0.60), likely due to the small salvage group (n=26) and limited overlap in the propensity score distribution. Consequently, the primary analysis relied on multivariable Cox regression rather than the matched cohort.

Abbreviations: SMD, standardized mean difference。

*EGFR*, epidermal growth factor receptor; *ALK*, anaplastic lymphoma kinase.

**Supplementary Table 2. Fine-Gray Competing Risk Analysis for Cumulative Incidence of Local Progression**

| **Time point**  **(months)** | **Consolidative SABR**  **(n=64)** | | **Salvage SABR**  **(n=26)** | |
| --- | --- | --- | --- | --- |
|  | **Local progression** | **Death** | **Local progression** | **Death** |
|  | CIF, % (95% CI) | CIF, % (95% CI) | CIF, % (95% CI) | CIF, % (95% CI) |
| 20 | 4.7 (0.0–9.9) | 9.4 (2.2–16.6) | 15.4 (1.2–29.6) | 11.5 (0.0–24.1) |
| 40 | 6.8 (0.2–13.5) | 35.1(21.0–49.3) | 15.4 (1.2–29.6) | 18.8 (0.4–37.3) |
| 60 | 10.1 (1.0–19.1) | 61.9 (32.0–91.8) | NR | NR |

Gray test for local progression: χ² = 1.40, P = 0.236.

Death was treated as a competing risk. The 60-month cumulative incidence estimates for the salvage group were not estimable due to zero patients at risk. The wide confidence intervals at 60 months reflect limited patient numbers at this time point.

Abbreviations:SABR, stereotactic body radiotherapy; CIF, cumulative incidence function; NR, not reached due to zero patients at risk.

**Supplementary Table 3. Fine-Gray Competing Risk Analysis for Cumulative Incidence of distant metastasis**

| **Time point (months)** | **Consolidative SABR**  **(n=64)** | **Salvage SABR**  **(n=26)** |
| --- | --- | --- |
|  | **CIF, % (95% CI)** | **CIF, % (95% CI)** |
| 10 | 25.0 (14.3–35.7) | 34.6 (15.9–53.4) |
| 20 | 36.2 (24.2–48.2) | 50.0 (30.2–69.8) |
| 30 | 46.5 (33.3–59.6) | 50.0 (30.2–69.8) |
| 40 | 57.8 (43.2–72.3) | 58.3 (35.1–81.5) |
| 50 | 57.8 (43.2–72.3) | 58.3 (35.1–81.5) |
| 60 | 57.8 (43.2–72.3) | NR |

Gray test for distant metastasis: χ² = 0.43, P = 0.513.

Note: Death was treated as a competing risk. The 60-month cumulative incidence of death was 9.3% (95% CI, 0.0–18.5%) in the consolidative group and was not estimable in the salvage group.

Abbreviations: SABR, stereotactic ablative radiotherapy; CIF, cumulative incidence function; NR, not reached due to zero patients at risk.

**Supplementary Table 4. Fine-Gray Competing Risk Analysis for Local Progression-Free Survival**

| **Variable** | **Subdistribution HR** | **95% CI** | **P value** |
| --- | --- | --- | --- |
| Consolidative SABR (yes vs. no) | 0.43 | 0.13–1.44 | 0.107 |
| *EGFR/ALK* (positive vs. negative) | 0.41 | 0.11–1.61 | 0.200 |
| Oligometastatic status (yes vs. no) | 0.39 | 0.08–1.82 | 0.230 |
| Best response (SD vs. PR) | 0.74 | 0.20–2.72 | 0.650 |
| BED10 | 0.99 | 0.94–1.03 | 0.540 |

Death was treated as a competing risk. The Fine-Gray subdistribution hazard model was adjusted for the same covariates as the primary Cox regression. The pseudo likelihood ratio test for the overall model was 6.08 on 5 degrees of freedom.

Abbreviations: HR, hazard ratio; CI, confidence interval; SABR, stereotactic body radiotherapy; *EGFR*, epidermal growth factor receptor; *ALK*, anaplastic lymphoma kinase; SD, stable disease; PR, partial response; BED10, biologically effective dose with α/β=10.
