# Supplementary material for: Consolidative versus salvage stereotactic ablative radiotherapy to the primary lung tumor in stage IV non–small cell lung cancer
Source: Front Oncol. 2026 Jul 15;16:1758011. doi: 10.3389/fonc.2026.1758011 (PMC13414212; doi:10.3389/fonc.2026.1758011)
Supplement: Supplementary file 2 [file Table2.docx]

**Supplementary Table 5. Exploratory univariate analysis of factors associated with grade ≥ 2 radiation pneumonitis.**

| **Characteristic** | **Grade 0–1 RP (n=79)** | **Grade ≥2 RP (n=11)** | **P value** |
| --- | --- | --- | --- |
| Tumor location | | | |
| Central | 33 (82.5) | 7 (17.5) | 0.124 |
| Peripheral | 46 (92.0) | 4 (8.0) |  |
| BED10 (Gy) | | | |
| Median (range) | 100.0 (61.6–132.0) | 100.4 (100.0–132.0) | 0.073 |
| Fraction size | | | |
| ≥10 Gy/fraction | 55 (87.3) | 8 (12.7) | 0.747 |
| <10 Gy/fraction | 24 (88.9) | 3 (11.1) |  |
| GTV (cc) | | | |
| Median (range) | 8.8 (0.8–92.7) | 12.7 (2.4–46.3) | 0.219 |
| SABR timing | | | |
| Consolidative SABR | 57 (89.1) | 7 (10.9) | 1.000 |
| Salvage SABR | 22 (84.6) | 4 (15.4) |  |

Data are presented as n (%) for categorical variables and median (range) for continuous variables. P values were calculated using Fisher’s exact test for categorical variables and the Mann–Whitney U test for continuous variables.

Abbreviations: RP, radiation pneumonitis; BED10, biologically effective dose with α/β=10; GTV, gross tumor volume; SABR, stereotactic ablative radiotherapy.
