# Supplementary figures and images for: Consolidative versus salvage stereotactic ablative radiotherapy to the primary lung tumor in stage IV non–small cell lung cancer
Source: Front Oncol. 2026 Jul 15;16:1758011. doi: 10.3389/fonc.2026.1758011 (PMC13414212; doi:10.3389/fonc.2026.1758011)

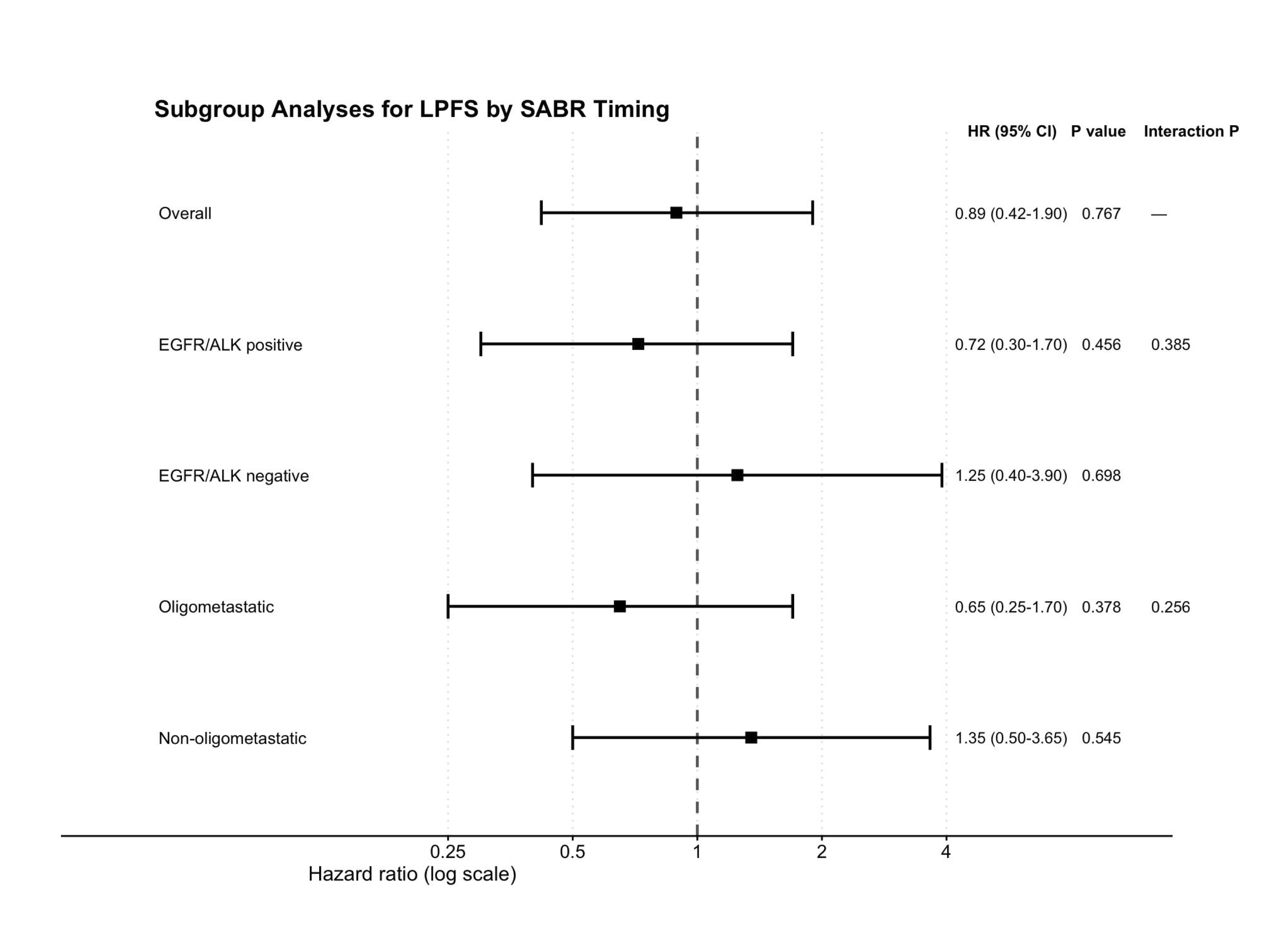

Supplement: Supplementary Table 1 — Propensity Score Matching (PSM) Balance Diagnostics. [file Image1.tiff]
